# Interleukin-10 enhances IgG galactosylation and sialylation

**DOI:** 10.1186/s40364-026-00966-4

**Published:** 2026-07-13

**Authors:** Hanna B. Lunding, Anna M. Wasynczuk, Yannic C. Bartsch, Jana Sophia Buhre, Jan Nouta, Alexei Leliavski, Selina Lehrian, Anna Emilia Becker, Kristina Manzhula, Philipp Köcher, Janina Mehlfeld, Johann Rahmöller, Manfred Wuhrer, Marc Ehlers

**Affiliations:** 1 Laboratory of Immunology, Institute of Nutritional Medicine, University of Luebeck and University Hospital Schleswig-Holstein, Campus Luebeck, Luebeck, Germany; 2 Center for Proteomics and Metabolomics, Leiden University Medical Center, Leiden, The Netherlands; 3 Laboratory of Anti-Viral Antibody-Omics, TWINCORE – Institute for Experimental and Clinical Infection Research, Helmholtz Center for Infection Research (HZI) and Medical School Hannover (MHH), Hannover, Germany; 4 Department of Anesthesiology and Intensive Care, University of Luebeck and University Hospital Schleswig-Holstein, Campus Luebeck, Luebeck, Germany; 5 Department of Anesthesiology, Haraldsplass Diakonale Sykehus, Bergen, Norway; 6 German Center for Lung Research (DZL), University of Luebeck, Luebeck, Germany

**Keywords:** Interleukin-10, B cell, Germinal center, IgG glycosylation, IgG galactosylation, IgG sialylation, T follicular cell, IFNγ, Plasma cell

## Abstract

IgG antibodies contain a conserved *N*-linked glycosylation site at Asn297 in the Fc region, and variations in Fc glycosylation critically influence antibody effector functions. While inflammatory signals during immunization are known to reduce IgG Fc galactosylation and sialylation, the counter-regulatory pathways that enhance these modifications remain poorly understood. Here, we investigated the association of the anti-inflammatory cytokine IL-10 with germinal center (GC) responses and IgG Fc glycosylation following protein immunization in mice. Blockade of IL-10 receptor signaling after immunization with a model protein and the adjuvant Alum reduced Fc galactosylation and sialylation of antigen-specific IgG1 and was associated with decreased expression of the sialyltransferase St6gal1 in antigen-specific GC B cells and plasma cells. Although IFNγ is known to suppress IgG Fc galactosylation and sialylation, Alum immunization paradoxically induced both high levels of Fc galactosylation and sialylation as well as a high frequency of IFNγ-producing CD4^+^ T follicular cells. Using IL-10 reporter mice, we resolved this apparent discrepancy by identifying a substantial population of IL-10⁺ IFNγ⁺ double-producing T follicular cells following Alum immunization. Ex vivo murine and human B cell culture experiments further showed that IL-10 counteracts IFNγ-mediated downregulation of St6gal1. Together, these findings identify an association between IL-10 signaling and increased St6gal1 expression in GC B cells and PCs, as well as increased IgG Fc galactosylation and sialylation. Furthermore, the data identify a population of IL-10⁺ IFNγ⁺ T follicular cells that may contribute to the IL-10-associated glycosylation changes. Our study supports a link between cytokine environments and IgG Fc glycosylation and provides a framework for future studies investigating how IL-10-associated pathways shape IgG glycosylation in vaccination and inflammatory disease settings.

**Supplementary Information:**

The online version contains supplementary material available at 10.1186/s40364-026-00966-4.

## Graphical Abstract

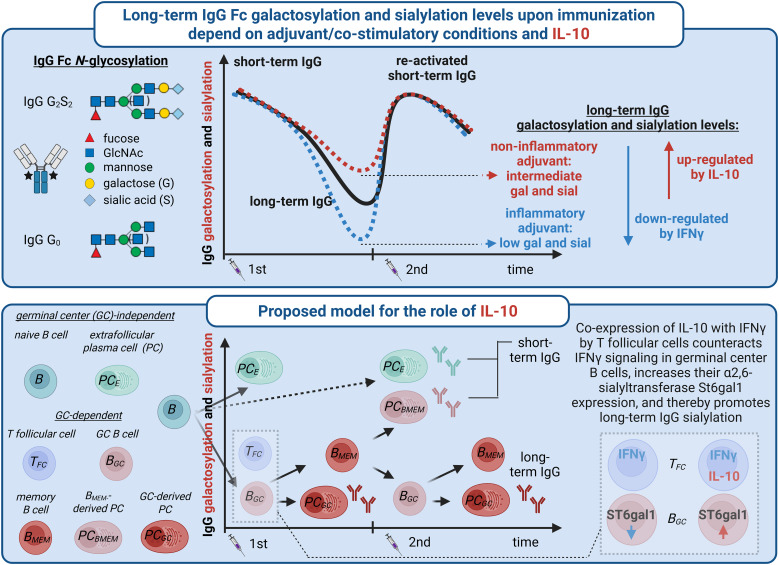

**To the editor**, 

IgG antibodies (Abs) contain a conserved *N*-linked glycosylation site at Asn297 within the Fc region (Fig. 1A). Different Fc glycosylation patterns originate primarily in Ab-producing plasma cells (PCs) due to co-stimulatory signals acting on differentiating B cells [1–5 and Suppl. Ref. E1]. These patterns have a critical influence on IgG effector functions [1, 6–9, E1-E4].

Recent studies in mice and humans suggest that short-term IgG responses after protein immunization with various adjuvants/co-stimuli, as well as re-activation of memory B cells immediately after booster immunization, are predominantly generated by extrafollicular PCs and exhibit high levels of Fc galactosylation and sialylation (Fig. 1B) [3, 5]. In contrast, more persistent long-term IgG responses that emerge later after immunization and are thought to arise predominantly from GC-derived PCs display lower levels of Fc galactosylation and sialylation (Fig. 1B) [3, 5].

In line with these observations, 15 days after immunization of mice with NP-CGG (4-hydroxy-3-nitrophenylacetyl–chicken gamma globulin) in complete Freund’s adjuvant (CFA), more than 80% of the antigen-specific PCs were already GC-derived (10).

Accordingly, a recurring IgG galactosylation and sialylation curve following each booster immunization was proposed [3, 5]. The enzymes responsible for adding galactose and sialic acid to newly generated IgG Abs in PCs are beta-1,4-galactosyltransferase (B4galt1) and alpha-2,6-sialyltransferase (St6gal1), respectively [4, E5]. Previous studies have suggested that their transcription is regulated by a shared gene cluster [11]. In addition, terminal galactose residues are required as substrates for subsequent sialylation.

Depending on the (inflammatory) potential of adjuvant-induced co-stimulatory signals, St6gal1 expression levels have been suggested to be primarily regulated at the level of IgG^+^ GC B cells and subsequently transmitted to their PC progeny [3].

Accordingly, immunization with the model protein ovalbumin (Ova) combined with the highly inflammatory *Mycobacterium tuberculosis* (*Mtb*)-enriched CFA (eCFA) induced lower levels of long-term anti-Ova IgG Fc galactosylation and sialylation upon day 14, compared to immunization with Ova, combined with the less inflammatory incomplete Freund’s adjuvant (IFA), and particularly with the least inflammatory adjuvant aluminum hydroxide (Alum) (Fig. 1B and C) [3]. Consistent with this pattern, St6gal1 protein expression in Ova-specific IgG^+^ GC B cells and PCs on day 12 was also lowest following Ova-eCFA immunization (Fig. 1C) [3]. Furthermore, two CD4^+^ T follicular cell subsets have been linked to the downregulation of St6gal1 in antigen-specific IgG^+^ GC B cells and their PC progenies, as well as to the generation of IgG Abs with low galactosylation and sialylation: IL-27-dependent IFNγ-producing and IL-23-dependent IL-17-producing T follicular cells [2, 3].

In contrast, the contribution of counter-regulatory signals that enhance long-term IgG Fc galactosylation and sialylation remains largely unexplored. Here, we investigated the role of the anti-inflammatory cytokine IL-10 in shaping long-term IgG Fc galactosylation and sialylation, as well as St6gal1 expression in GC and PC responses.

To assess the role of IL-10 in long-term IgG Fc glycosylation, mice were immunized with Ova-Alum, either with or without an anti-IL-10 receptor (IL-10R) blocking Ab administered three days after immunization to preferentially target the GC response (Fig. 1D and Suppl. M&M). Immune responses were analyzed 14 days after Ova-Alum immunization. The IL-10R blockade reduced long-term anti-Ova IgG1 Fc galactosylation and sialylation levels (Fig. 1E and Suppl. Tables S1 and S2). In addition, IL-10R blockade decreased St6gal1 expression in Ova-specific IgG1^+^ GC B cells and PCs (Fig. 1F–H).

**Fig. 1:**
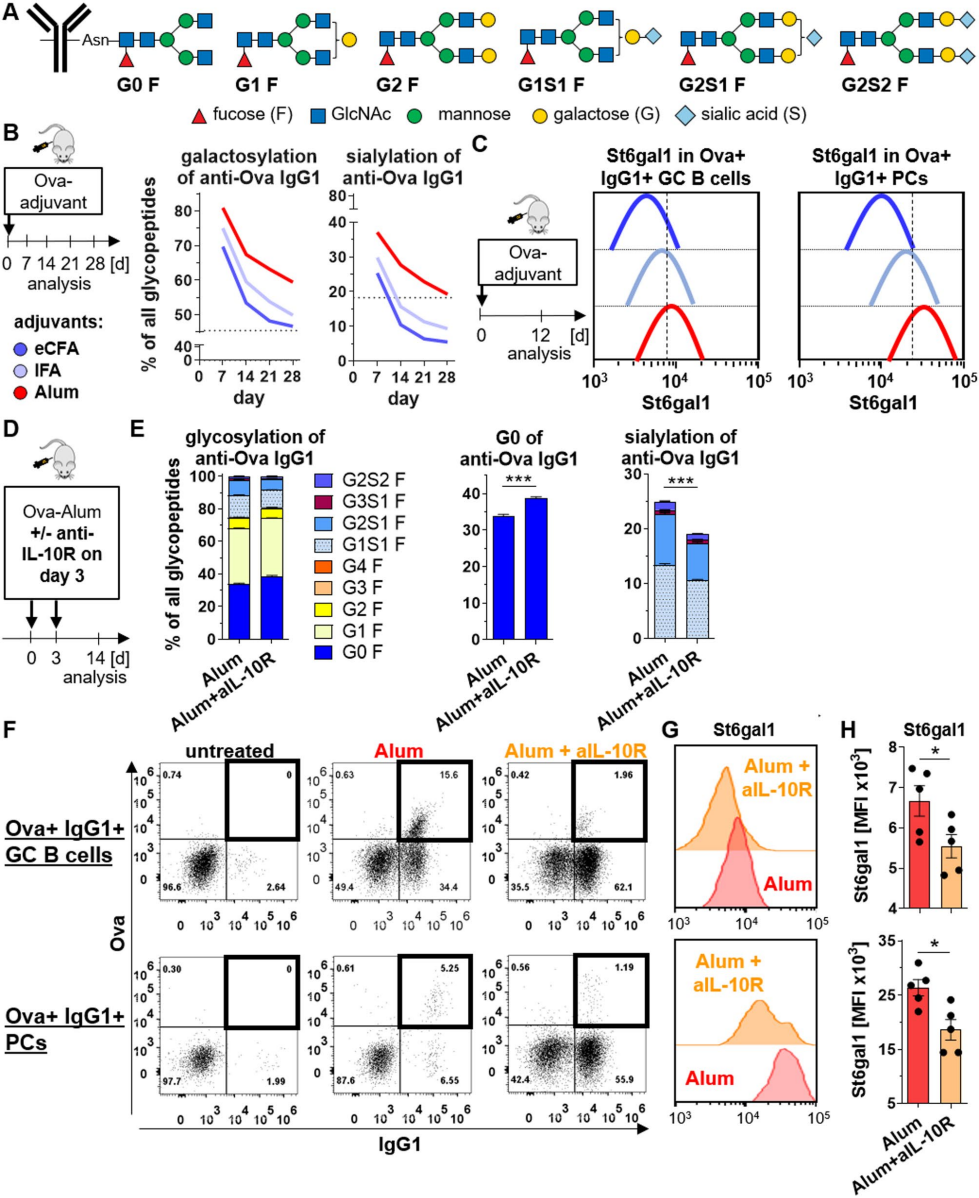
IL-10 enhances IgG Fc galactosylation and sialylation. (**A**) The six major glycans attached to Asn297 in the Fc part of murine IgG Abs. (**B**) Simplified presentation of previously published data (3). C57BL/6 wild-type (WT) mice were immunized intraperitoneally (i.p.) with Ova together with the adjuvants eCFA, IFA, or Alum. Serum anti-Ova IgG1 Fc galactosylation and sialylation were analyzed on days 7, 14, 21 and 28. Dotted lines indicate the average galactosylation and sialylation levels of bulk serum IgG1 Fc in untreated mice. (**C**) Simplified presentation of previously published data (3). C57BL/6 WT mice were immunized i.p. with Ova together with eCFA, IFA, or Alum and analyzed on day 12 for St6gal1 protein expression in Ova-specific IgG1+ splenic GC B cells (B220+ CD138− FAS+ GL-7+) and PCs (B220low/inter CD138+). Simplified St6gal1 expression is shown. Dotted lines represent the average St6gal1 expression in the corresponding IgG1+ cells of untreated mice. (**D**–**H**) (D) Experimental design: C57BL/6 WT mice were immunized i.p. with Ova and Alum on day 0, with or without an anti-IL-10R blocking Ab administered on day 3 (n = 5 per group). The mice were analyzed on day 14 for: (E) serum anti-Ova IgG1 Fc glycosylation, including agalactosylation (G0), and accumulated sialylation (G1S1F + G2S1F + G3S1F + G2S2F), assessed by nLC-MS (percentages, mean ± SEM); and (**F**–**H**) median fluorescence intensity (MFI) of St6gal1 protein expression in Ova-specific IgG1+ splenic GC B cells and PCs measured by flow cytometry. (F) Gating strategy for Ova-specific IgG1+ GC B cells and PCs (highlighted gates in representative plots). (G) Representative histograms showing St6gal1 expression in Ova-specific IgG1+ cells from individual mice. (H) Quantification (mean ± SEM) of St6gal1 MFI of all mice. Significances in the figure parts are based on two-tailed t-tests (*p < 0.05, **p < 0.01, ***p < 0.001). The Shapiro–Wilk test revealed no significant deviation from normal distribution in any of the datasets. One of two independent experiments is shown

Although it cannot be ruled out that IL-10R blockade affects early extrafollicular PC responses, these findings are consistent with an association between an IL-10-dependent increase in galactosylation and sialylation of long-term, antigen-specific IgG1 and increased St6gal1 expression in IgG1^+^ GC B cells and PCs after Ova-Alum immunization. This is further supported by a strong correlation between IgG1 Fc galactosylation and sialylation levels and St6gal1 expression in GC B cells and PCs across all individual mice (Suppl. Fig. S1).

As previously shown, immunization of *Ifngr1*-deficient mice, which lack the IFNγ receptor 1 subunit, with Ova-CFA or Ova-eCFA led to higher levels of long-term anti-Ova IgG1 and IgG2 Fc galactosylation and sialylation compared with wild-type controls, indicating the critical role of IFNγ receptor signaling in downregulating long-term IgG Fc galactosylation and sialylation (Fig. 2A and Suppl. Fig. S2) [3, E1]. Surprisingly, Ova-Alum induced the highest frequency of IFNγ-producing CD4^+^ T follicular cells on day 12 compared to Ova-IFA and Ova-eCFA, despite resulting in the highest anti-Ova IgG galactosylation and sialylation levels (Fig. 2B).

As the co-expression of IL-10 and IFNγ in CD4^+^ type 1 regulatory T cells has been linked to an inhibitory function, in contrast to IFNγ-only-producing CD4^+^ T cells [12, E6], we hypothesized that IL-10 may also be co-expressed with IFNγ in CD4^+^ T follicular cells to modulate GC B cell responses. Analysis of IL-10 reporter mice 12 days after immunization revealed that Ova-Alum induced the highest frequency and total numbers of IL-10^+^ IFNγ^+^ T follicular cells (Fig. 2C-E and Suppl. Fig. S3). Consistently, IL-10 counteracted the IFNγ-induced downregulation of St6gal1 in LPS-stimulated murine splenic B cells and of ST6GAL1 in R848-stimulated human peripheral blood mononuclear cells (PBMCs) ex vivo (Fig. 2F –H and Suppl. Fig. S4).

Together, these data are consistent with a model in which IL-10 signaling is associated with increased St6gal1 expression in GC B cells and PCs and enhanced long-term IgG Fc galactosylation and sialylation following Alum immunization. The observed enrichment of IL-10⁺ IFNγ⁺ T follicular cells following Alum immunization raises the possibility that IL-10 produced by these cells may counteract IFNγ-mediated suppression of St6gal1 in IgG^+^ GC B cells and their PC progeny, thereby enhancing galactosylation and sialylation of long-term IgG (Fig. 2I). That IL-10 can counteract IFNγ-mediated downregulation of St6gal1 was supported by the B cell culture data.

**Fig. 2:**
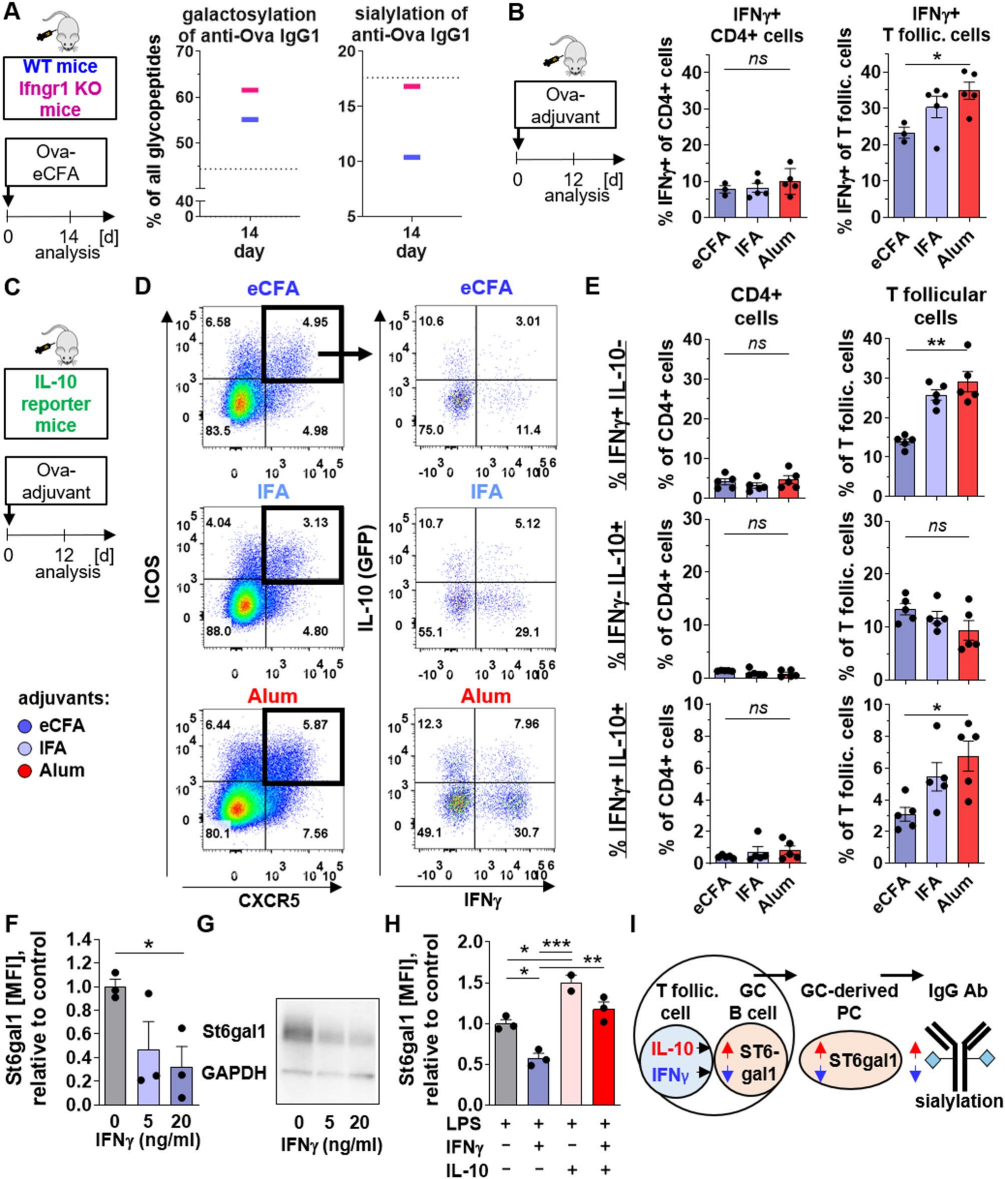
IL-10 counteracts IFNγ-induced St6gal1 down-regulation. (**A**) Simplified presentation of previously published data (3). C57BL/6 WT and Ifngr1-KO mice were immunized i.p. with Ova together with the adjuvant eCFA and analyzed on day 14 for serum anti-Ova IgG1 Fc glycosylation. Anti-Ova IgG1 Fc galactosylation and sialylation are shown. Dotted lines represent the average galactosylation and sialylation levels of bulk serum IgG1 Fc in untreated mice. (**B**) Experimental design: C57BL/6 WT mice were immunized i.p. with Ova together with the indicated adjuvants (n = 3–5 per group). On day 12, the frequencies of IFNγ+ CD4+ T cells and IFNγ+ CD4+ ICOS+ CXCR5+ T follicular cells were analyzed. Data are shown as means ± SEM. One of two independent experiments is shown. (**C**–**E**) (C) Experimental design: IL-10 reporter (GFP; green fluorescent protein) mice were immunized i.p. with Ova together with the indicated adjuvants, and their spleen cells were analyzed on day 12 by flow cytometry (n = 5 per group). (D) Representative expression of IFNγ and IL-10 in CD4+ CXCR5+ ICOS+ T follicular cells (gated populations shown in bold squares in representative plots) derived from previously gated CD4+ cells. (E) Frequencies of (i) IFNγ+ IL-10−, (ii) IFNγ− IL-10+, and (iii) IFNγ+ IL-10+ cells within CD4+ T cells (gating not shown) and within CD4+ ICOS+ CXCR5+ T follicular cells (gating shown in D), shown as mean ± SEM across all mice. One of two independent experiments is shown. (**F**–**H**) Experimental design: enriched splenic murine B cells from untreated mice were stimulated with LPS and the indicated concentrations of IFNγ (± 20 ng IFNγ± 10 ng IL-10 in (H)) and analyzed on day 4. (F and H) Median fluorescence intensity (MFI) of St6gal1 protein expression in B220+ cells (gating not shown) measured by flow cytometry (n = 3 per group). Data are shown as mean ± SEM and were normalized to the LPS-only group. (G) St6gal1 and GAPDH protein expression in cultured cells determined by Western blotting; pooled samples from each group were loaded. One of three independent experiments is shown in (F), (G) and (H). (**I**) Proposed model summarizing the findings of this study. See text for details. Non-parametric Kruskal-Wallis tests with Dunn's post hoc multiple comparisons tests were used for (B), (E) and (F). Two-way ANOVA with Tukey’s post hoc multiple-comparisons test was used for (H) because two (cytokine) factors were analyzed. *p < 0.05, **p < 0.01, ***p < 0.001

Several limitations should be considered. IL-10R blockade was performed systemically and therefore may have affected multiple immune cell populations. Furthermore, although IL-10⁺ IFNγ⁺ T follicular cells were associated with conditions promoting increased IgG galactosylation and sialylation, the present study does not establish a causal role for this subset. Likewise, whether IL-10 acts directly on GC B cells and their plasma cell progeny, indirectly through other immune cell populations, and also on extrafollicular B cell responses was not addressed. In addition, other cytokines and glycosyltransferases as well as independent regulation of the B4galt1, not examined here, may contribute to the observed changes in antibody glycosylation.

The functional implications of antigen-specific IgG Abs with differential Fc galactosylation and sialylation following immunization and booster immunization remain incompletely understood. The following hypotheses integrate prior literature with observations from the present study and should be regarded as speculative models that may help contextualize our findings. Early strong levels of highly galactosylated and sialylated short-term IgG Abs, most likely generated by extrafollicular PC responses [3, 5, E7], may favor complement activation via C1q and affinity maturation in GCs [E3-E4]. In contrast, when IgG levels are low in the long term and the neutralizing capacity is low, immune complexes formed upon new antigen contact may be advantageous for antigen uptake by dendritic cells and monocytes, as well as re-activation of memory B cells and subsequent immune response, if the long-term IgGs are lowest galactosylated and sialylated [3, 5, E7]. This may be explained by inhibitory effects attributed to galactosylated or sialylated short-term as well as long-term IgG on these cells, most likely through interaction with inhibitory receptors recognizing IgG Fc glycan structures [1, 7, 9, E1-E2].

Adjuvants/co-stimuli as well as gene polymorphisms that promote IL-10 production may counteract the generation of agalactosylated long-term IgG Abs, thereby inhibiting effective long-term IgG responses to re-activate immune responses. Future studies will be required to determine whether such IL-10-associated glycosylation changes have implications for vaccine-induced immunity or inflammatory autoimmune diseases characterized by increased levels of agalactosylated IgG [6, 7, 13, E2, E8].

In summary, our findings identify an association between IL-10 signaling, increased St6gal1 expression in GC B cells and PCs, and enhanced IgG Fc galactosylation and sialylation following immunization. In addition, we identify a population of IL-10⁺ IFNγ⁺ T follicular cells that may contribute to the cytokine milieu associated with these glycosylation changes.

## Supplementary Information

Below is the link to the electronic supplementary material.

Supplementary Material 1

## Data Availability

All data are presented in the main manuscript or in the Supplementary Data.

## Abbreviations

Ab: Antibody
Alum: Aluminum hydroxide
CFA: Complete Freund’s adjuvant
eCFA: Mycobacterium tuberculosis (Mtb)-enriched complete Freund’s adjuvant
GC: Germinal center
IFA: Incomplete Freund’s adjuvant
IgG: Immunoglobulin G
IL: Interleukin
IL-10R: IL-10 receptor
MFI: Median fluorescence intensity
Ova: Ovalbumin
PC: Plasma cell
