# Supplementary material for: Interleukin-10 enhances IgG galactosylation and sialylation
Source: Biomark Res. 2026 Jul 13;14:82. doi: 10.1186/s40364-026-00966-4 (PMC13366667; doi:10.1186/s40364-026-00966-4)
Supplement: Supplementary file 1 — Supplementary Material 1 [file 40364_2026_966_MOESM1_ESM.pdf]

Supplementary Information

Interleukin-10 enhances IgG galactosylation and sialylation

Hanna B. Lunding[1], Anna M. Wasynczuk[2], Yannic C. Bartsch[1,3], Jana Sophia Buhre[1], Jan Nouta[2], Alexei Leliavski[1], Selina Lehrian[1], Anna Emilia Becker[1], Kristina Manzhula[1], Philipp Köcher[1], Janina Mehlfeld[1], Johann Rahmöller[1,4.5], Manfred Wuhrer[2,*], Marc Ehlers[1,6,*]

Supplementary Material & Methods

Mice

C57BL/6 wild-type (WT) mice were purchased from The Jackson Laboratory and bred in our animal facility. Additional C57BL/6 WT mice were obtained from Janvier Labs (Le Genest Saint-Isle, France). *Ifngr1*-knock-out (KO) mice lacking the ligand-binding IFNγ receptor 1 were generated as described and backcrossed for at least eight generations onto the C57BL/6 background (E9). IL-10 reporter (Vert-X) mice were originally provided by Christopher L. Karp (Cincinnati Children's Hospital Medical Center, Cincinnati, Ohio, USA) (E10) and were bred in-house after backcrossing for at least eight generations onto the C57BL/6 background. Mice were housed and bred in the animal facility of the University of Luebeck under specific pathogen-free conditions at 22 °C with a 12-hour light–dark cycle and had ad libitum access to food and water. Sex-matched, 10–12-week-old mice were used for all experiments.

Ethical considerations

All mouse experiments were performed in accordance with the German animal welfare legislation and were approved by the relevant institutional animal care and use committee.

Human blood samples were obtained after written informed consent and approval by the Ethics Committee of the University of Lübeck, Germany (approval no. 20-123).

Reagents

Ovalbumin (Ova, grade VI, #A2512) and incomplete Freund's adjuvant (IFA, #F5506) were purchased from Sigma-Aldrich (St. Louis, MO, USA). Mycobacterium tuberculosis (Mtb)-enriched

complete Freund's adjuvant (eCFA) was prepared by adding heat-killed Mtb H37RA (BD Biosciences (San Diego, CA, USA) to IFA at a concentration of 5 mg/mL (corresponding to a five-fold higher concentration than in commercially available complete Freund's adjuvant (CFA, 1 mg/mL). Aluminum hydroxide (Alum) was purchased from InvivoGen (Alhydrogel adjuvant 2%, #vac-alu, Toulouse, France).

Ovalbumin immunizations

C57BL/6 mice were immunized intraperitoneally (i.p.) with 100 µg of Ova protein together with the indicated adjuvants (Alum or the more inflammatory water-in-oil adjuvants IFA and eCFA) in a total volume of 200 µL. Immunizations were prepared by mixing 100 µL of an Ova-PBS solution (1 mg/mL) with 100 µL of the respective adjuvant solution (Alum, IFA, or eCFA) prior to immunization. The dosing was based on prior experiments (E12 (3)). Serum and splenocytes were collected on the indicated days. Serum samples were stored at −20 °C, whereas splenocytes were kept on ice and analyzed by flow cytometry on the same day. Where indicated, 240 µg of an anti-IL-10 receptor (IL-10R) rat IgG1 monoclonal Ab (clone 1B1.3A, #BP0050, Bio X cell) in 200 µL PBS was administered i.p three days after Ova immunization. No formal batch correction was applied, as all samples within each experiment were processed and analyzed under identical experimental conditions. Formal blinding was not performed. Samples were analyzed using predefined gating and analysis strategies that were applied consistently across experimental groups.

Analysis of IgG subclass Fc glycosylation via nano liquid chromatography-mass spectrometry (nLC-MS)

Murine IgG Fc subclass glycopeptides were analyzed by nLC-MS as described previously (E11-E13). In brief, bulk serum IgGs were affinity-captured using Protein G Sepharose (GE Healthcare). Anti-Ova Abs were affinity-captured either using Ova-coupled Sepharose or in-house generated Ova-coated plates (Thermo Fisher Scientific, Roskilde, Denmark). Abs were eluted with 100 mM formic acid. Eluates were dried using vacuum centrifugation, subjected to tryptic cleavage, and subsequently analyzed by nLC-MS. Peak areas of IgG1- and IgG2-specific Fc *N*-glycopeptides were background-corrected and assigned to one of nine glycan compositions: G0, G1, G2, G3, G4, G1S1, G2S1, G3S1, and G2S2 (**Suppl. Table S1**). IgG2c (the C57BL/6 polymorphic variant of IgG2a) glycoforms were not separated from IgG2b glycoforms and were therefore quantified together. The relative intensity of each IgG1 Fc glycopeptide was calculated by normalizing the glycopeptide intensity to the sum intensity of all IgG1 glycopeptides (total area normalization). Site-specific total area normalization is commonly performed in glycosylation analysis of

immunoglobulins, as it reflects aspects of the biosynthetic process such as diversification of the glycosylation profile along the biosynthetic path, with possible synergies and competition between glycosylation events occurring on a specific site. These values were then used to calculate glycosylation traits: IgG1 G0 (agalactosylation), IgG1 galactosylation, and IgG1 sialylation (**Suppl. Table S2**). Low abundance signals corresponding to IgG1 G3F, G4F, and G3S1F glycopeptides carrying additional alpha-1,3-linked terminal galactose(s) were detected at relative intensities below 3%. IgG1 glycopeptides containing bisecting N-acetylglucosamine (GlcNAc) or lacking fucose (F0) were not detected, or were below limit of quantification. The intensities of anti-Ova IgG2 (IgG2c + IgG2b) induced by Ova-Alum were too low for quantification.

Flow cytometric analysis of murine cells

Spleen cells from untreated and immunized mice, or cultured splenic B cells, were passed through a sieve and prepared for flow cytometric measurement using an LSRII (BD Biosciences) or an Attune NxT (Thermo Fisher Scientific).

For surface staining, cells were incubated at 4 °C for 1 h with the following biotin- or fluorochrome-conjugated Abs and molecules: anti-B220 (clone RA3-6B2, BD Biosciences), anti-CD95 (FAS, Jo-2, BD Biosciences), anti-CD138 (281-2, BD Biosciences), anti-IgG1 (RMG1-1, BioLegend), anti-CD4 (RM4-5, BioLegend), anti-CXCR5 (L138D7, BioLegend), anti-GL-7 (GL-7, BioLegend), anti-ICOS (C398.4A, BioLegend), Ova (Thermo Fisher Scientific) and a viability dye. For subsequent intracellular staining, surface-stained samples were fixed with Cytofix/Cytoperm (#554722, BD Biosciences) according to the manufacturer's instructions, permeabilized using a buffer containing 0.05% saponin in 0.05× PBS, and stained at 4 °C for 1 h. The following biotin- or fluorochrome-conjugated Abs and molecules were used for intracellular staining: anti-IFNγ (clone XMG1.2, BD Biosciences), anti-IgG1 (RMG1-1, BioLegend), anti-St6gal1 (polyclonal goat IgG Ab, R&D Systems) or corresponding goat IgG isotype control (R&D Systems), streptavidin (BioLegend) and Ova (Thermo Fisher Scientific). For extra- and intracellular staining, Abs were premixed at a final concentration of 1 µg/mL each. When biotinylated Abs were used, an additional staining step with fluorochrome-coupled streptavidin (BioLegend) was performed at 4 °C for 1 h. For intracellular cytokine analysis in T cells, cells were stimulated prior to staining with a cell stimulation cocktail containing phorbol myristate acetate (PMA) ionomycin, and the protein transport inhibitors brefeldin A and monensin (#00-4975-93, eBioscience) according to the manufacturer's instructions.

Flow cytometry data were analyzed using FlowJo v10.9.0 (BD Biosciences). To analyze St6gal1 protein expression in Ova-specific IgG1^{+} splenic GC B cells and PCs, the following gating strategy was applied: (i) lymphocytes were gated in FSC/SSC, (ii) singlets were selected in FSC-

A/FSC-H, (iii) live cells were gated, (iv) B220 versus CD138 was used to define PCs ($B220^{low/inter}$ $CD138^{+}$) and non-PC B cells ($B220^{+}$ $CD138^{-}$), (v) non-PC B cells were further gated for GC B cells (FAS^{+} GL-7^{+}), (vi) GC B cells ($B220^{+}$ $CD138^{-}$ FAS^{+} GL-7^{+}) and PCs ($B220^{low/inter}$ $CD138^{+}$) were analyzed for IgG1 expression versus Ova-binding, (vii) and to St6gal1 expression was quantified as median fluorescence intensity (MFI)) in Ova-specific $IgG1^{+}$ cells.

To analyze IFNγ and/or IL-10 expression in $CD4^{+}$ T cells and T follicular cells, the following gating strategy was used: (i) lymphocytes were gated in FSC/SSC, (ii) singlets were selected, (iii) live cells were gated, (iv) $CD4^{+}$ T cells were identified, (v) $CXCR5^{+}$ $ICOS^{+}$ T follicular cells were gated from $CD4^{+}$ T cells. IFNγ expression, or IFNγ in combination with IL-10 (GFP^{+} in the IL-10 reporter mice), was analyzed in $CD4^{+}$ T cells and $CD4^{+}$ $CXCR5^{+}$ $ICOS^{+}$ T follicular cells.

The gating strategies were applied consistently across all samples within each experiment, and representative gating plots have been included in the figures.

Murine B cell culture

Murine splenic B cells of untreated mice were isolated using a mouse B cell isolation kit (Miltenyi Biotec) via negative selection, resulting in > 95% purity ($B220^{+}$ cells). Enriched B cells ($6–8 \times 10^{4}$ cells/mL) were cultured in RPMI-1640 medium supplemented with L-glutamine (Thermo Fisher Scientific), 10% fetal calf serum (FCS, Gibco, Thermo Fisher Scientific), 50 µM 2-mercaptoethanol (Sigma-Aldrich), 1 mM HEPES (Sigma-Aldrich), and 100 U/mL penicillin/streptomycin (Gibco, Thermo Fisher Scientific) at 37 °C and 5% CO_2. Cells were stimulated with 2.5 µg/mL LPS (from *E. coli* 0111:B4, Sigma-Aldrich) and, where indicated, with 5 or 20 ng/mL recombinant murine IFNγ (#315-05, Peprotech), and/or with 10 ng/mL recombinant murine IL-10 (#210-10, Peprotech). On day 4, St6gal1 protein expression (median fluorescence intensity, MFI) in gated $B220^{+}$ live cells was analyzed by flow cytometry. Cells were surface-stained with anti-B220 and subsequently stained intracellularly with anti-St6gal1 (see above). Alternatively, cultured cells were analyzed by Western blotting.

Human B cell culture and analysis

Human peripheral blood mononuclear cells (PBMCs) were isolated from the venous blood samples taken from healthy donors using density-gradient centrifugation with BioColl (density: 1.077 g/mL, Bio&Sell). The isolated PBMCs (1×10^{6} cells/mL) were cultured in RPMI-1640 medium supplemented with L-glutamine (Thermo Fisher Scientific), 10% FCS (FBS Advanced, #FBS-11A, Capricorn Scientific, Ebsdorfergrund, Germany), 50 µM 2-mercaptoethanol (Sigma-Aldrich), and 100 U/mL penicillin/streptomycin (Gibco, Thermo Fisher Scientific), at 37 °C and

5% CO_2. The cells were stimulated with 1 µg/mL Resiquimod (R848, Enzo Life Sciences GmbH) and, where indicated, with 10 ng/mL recombinant human IFN-γ (#300-02, PeproTech), and/or with 100 ng/mL recombinant murine IL-10 (#210-10, PeproTech). The rationale behind the selection of R848 as a ground survival and proliferation stimulus for the human B cell culture is predicated on the finding that human cultured B cells were found to be incompatible with LPS, resulting in their inability to survive. On day 5, St6gal1 protein expression (MFI) in gated $CD38^+$, $CD38^+$ IgG^+ and $CD38^+$ IgA^+ live PCs was analyzed by flow cytometry.

Flow cytometric measurements were performed using an Attune NxT (Thermo Fisher Scientific). Cultured human PBMCs (5×10^5 cells/100µL) were stained for surface markers at 4 °C for 1 h using fluorochrome-conjugated Abs against CD38 (#303526, BioLegend), IgG (#410740, BioLegend), and IgA (#130-113-477, Miltenyi Biotec), along with a viability dye (#L34976, Life Technologies GmbH). The cells were then fixed and permeabilized using Cytofix/Cytoperm (#554722, BD Biosciences) according to the manufacturer's instructions, and incubated at 4 °C overnight (16 h) with anti-St6gal1 (polyclonal goat IgG Ab, R&D Systems), as well as Abs against IgG and IgA. For extra- and intracellular staining, Abs were premixed at a final concentration of 1 µg/mL each.

Flow cytometry data were analyzed using FlowJo v10.9.0 (BD Biosciences). To quantify St6gal1 protein expression (MFI) in $CD38^+$, $CD38^+$ IgG^+ and $CD38^+$ IgA^+ PCs, the following gating strategy was applied: (i) lymphocytes were gated in FSC/SSC, (ii) singlets were selected in FSC-A/FSC-H, (iii) live cells were gated, and (iv) CD38, IgG, and IgA were used to identify $CD38^+$ PCs, IgG^+ $CD38^+$ PCs and IgA^+ $CD38^+$ PCs.

Western blotting

Enriched B cells were cultured for 4 days as described above. Cells from parallel wells 2×10^6 were pooled, pelleted, and lysed in RIPA buffer (150 mM NaCl, 1% NP-40, 0.1% SDS, 50 mM Tris, and 0.5% sodium deoxycholate) supplemented with a protease inhibitor cocktail (Roche). Protein concentration was determined using a Pierce™ BCA Protein Assay Kit (Thermo Fisher Scientific). Equal amounts of protein (2 µg) were mixed with reducing buffer, separated by SDS-PAGE, and transferred to a polyvinylidene difluoride (PVDF) membrane using the Trans-Blot Turbo Transfer System (Bio-Rad). Membranes were blocked with 5% non-fat milk in TBS-Tween 20, and incubated with primary Abs against St6gal1 (polyclonal goat IgG, #AF5924, R&D Systems) and GAPDH (#AF5718, R&D Systems). Detection was performed using HRP-conjugated anti-goat secondary Abs and Immobilon Western HRP substrate (#WBKLS0050, Millipore).

Statistical analysis

Statistical analyses were performed using GraphPad Prism v10.0 (GraphPad Software, La Jolla, California, USA). Specific statistic analyses are described in the figure legends.

Supplementary References

E1. Hess C, Winkler A, Lorenz AK, Holecska V, Blanchard V, Eiglmeier S, Schoen A-L, Bitterling J, Stoehr AD, Petzold D, Schommartz T, Mertes MMM, Schoen CT, Tiburzy B, Herrmann A, Köhl J, Manz RA, Madaio MP, Berger M, Wardemann H, and Ehlers M. T cell-independent B cell activation induces immunosuppressive sialylated IgG antibodies. J Clinical Invest 2013; 123(9):3788–3796. doi: 10.1172/JCI65938. PMID: 23979161.

E2. Bartsch Y, Rahmöller J, Mertes MMM, Eiglmeier S, Lorenz FKM, Stoehr AD, Braumann D, Lorenz AK, Winkler A, Lilienthal G-M, Petry J, Hobusch J, Steinhaus M, Hess C, Holecska V, Schoen CT, Oefner CM, Leliavski A, Blanchard V, and Ehlers M. Sialylated autoantigen-reactive IgG antibodies attenuate disease development in autoimmune mouse models of lupus nephritis and rheumatoid arthritis. Front Immunol 2018; 9:1183. doi: 10.3389/fimmu.2018.01183. PMID: 29928274.

E3. Wang TT, Maamary J, Tan GS, Bournazos S, Davis CW, Krammer F, Schlesinger SJ, Palese P, Ahmed R, Ravetch JV. Anti-HA Glycoforms Drive B Cell Affinity Selection and Determine Influenza Vaccine Efficacy. Cell. 2015; 162(1):160-9. doi: 10.1016/j.cell.2015.06.026. PMID: 26140596.

E4. Lofano G, Gorman MJ, Yousif AS, Yu WH, Fox JM, Dugast AS, Ackerman ME, Suscovich TJ, Weiner J, Barouch D, Streeck H, Little S, Smith D, Richman D, Lauffenburger D, Walker BD, Diamond MS, Alter G. Antigen-specific antibody Fc glycosylation enhances humoral immunity via the recruitment of complement. Sci Immunol. 2018; 3(26):eaat7796. doi: 10.1126/sciimmunol.aat7796. PMID: 30120121.

E5. Sha J, Zhang R, Fan J, Gu Y, Pan Y, Han J, Xu X, Ren S, Gu J. The B-cell-specific ablation of B4GALT1 reduces cancer formation and reverses the changes in serum IgG glycans during the induction of mouse hepatocellular carcinoma. Cancers (Basel) 2022; 14(5):1333. doi: 10.3390/cancers14051333. PMID: 35267641.

E6. Pot C, Apetoh L, Kuchroo VK. Type 1 regulatory T cells (Tr1) in autoimmunity. Semin Immunol 2011; 23(3):202-8. Review. doi: 10.1016/j.smim.2011.07.005. PMID: 21840222.

E7. Buhre JS, Pongracz T, Künsting I, Lixenfeld AS, Wang W, Nouta J, Lehrian S, Schmelter F, Lunding HB, Dühring L, Kern C, Petry J, Martin EL, Föh B, Steinhaus M, von Kopylow V, Sina C, Graf T, Rahmöller J, Wuhrer M, Ehlers M. mRNA vaccines against SARS-CoV-2 induce comparably low long-term IgG Fc galactosylation and sialylation levels but increasing long-term IgG4 responses compared to an adenovirus-based vaccine. Front Immunol 2023; 13:1020844. doi: 10.3389/fimmu.2022.1020844. PMID: 36713457.

E8. Ohmi Y, Ise W, Harazono A, Takakura D, Fukuyama H, Baba Y, Narazaki M, Shoda H, Takahashi N, Ohkawa Y, Ji S, Sugiyama F, Fujio K, Kumanogoh A, Yamamoto K, Kawasaki N, Kurosaki T, Takahashi Y, Furukawa K. Sialylation converts arthritogenic IgG into inhibitors of collagen-induced arthritis. Nat Commun 2016; 7:11205. doi: 10.1038/ncomms11205. PMID: 27046227.

E9. Huang S, Hendriks W, Althage A, Hemmi S, Bluethmann H, Kamijo R, Vilcek J, Zinkernagel RM, Aguet M. Immune response in mice that lack the interferon-gamma receptor. Science 1993; 259(5102):1742-5. doi: 10.1126/science.8456301. PMID: 8456301.

E10. Madan R, Demircik F, Surianarayanan S, Allen JL, Divanovic S, Trompette A, et al. Nonredundant roles for B cell-derived IL-10 in immune counter-regulation. J Immunol 2009; 183: 2312–2320. doi:10.4049/jimmunol.0900185. PMID: 19620304.

E11. de Haan N, Reiding KR, Krištić J, Hipgrave Ederveen AL, Lauc G, Wuhrer M. The *N*-glycosylation of mouse immunoglobulin G (IgG)-fragment crystallizable differs between IgG subclasses and strains. Front Immunol. 2017; 8:608. doi: 10.3389/fimmu.2017.00608. PMID: 28620376.

E12 (3). Bartsch YC, Eschweiler S, Leliavski A, Lunding H, Wagt S, Petry J, Lilienthal G-M, Rahmöller J, de Haan N, Hölscher A, Erapaneedi R, Giannou AD, Aly L, Sato R, de Neef LA, Winkler A, Braumann D, Hobusch J, Kuhnigk K, Krémer V, Steinhaus M, Blanchard V, Gemoll T, Habermann J, Collin M, Salinas-Riesters G, Manz R, Korn T, Fukuyama H, Waisman A, Yogev N, Huber S, Rabe B, Rose-John S, Busch H, Berberich-Siebelt F, Hölscher C, Wuhrer M, and Ehlers M. IgG Fc sialylation is regulated during the germinal center reaction upon immunization with different adjuvants. J Allergy Clin Immunol 2020; 146(3):652-666. doi: 10.1016/j.jaci.2020.04.059. PMID: 32445838.

E13. Falk D, Wuhrer M. GlYcoLISA: antigen-specific and subclass-specific IgG Fc glycosylation analysis based on an immunosorbent assay with an LC-MS readout. Nat Protoc 2024 Jun;19(6):1887-1909. doi: 10.1038/s41596-024-00963-7. PMID: 38383719.

Supplementary Tables and Figure

Supplementary Table S1: IgG glycoforms. Abbreviation and proposed structure of the identified IgG subclass Fc-glycoforms.

Glycoform	Structure	IgG1 and IgG2 (IgG2c+b)
G0F	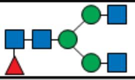	x
G1F	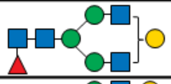	x
G2F	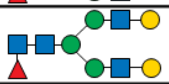	x
G3F	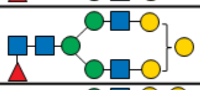	x
G4F	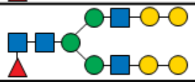	x
G1S1F	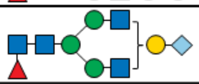	x
G2S1F	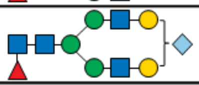	x
G3S1F	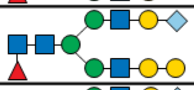	x
G2S2F	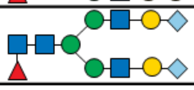	x

Supplementary Table S2: Description and formula for the calculation of the IgG subclass glycosylation traits.

Glycosylation trait	Description	Formula (based on Supplementary Table S1)
**galactosylation**	N-glycans carrying a galactose	(G1F + G2F + G3F + G4F + G1S1F + G2S1F + G3S1F + G2S2F) / sum of all IgG subclass glycopeptides
**sialylation**	N-glycans carrying an N-glycolylneuraminic acid (sialic acid)	(G1S1F + G2S1F + G3S1F + G2S2F) / sum of all IgG subclass glycopeptides

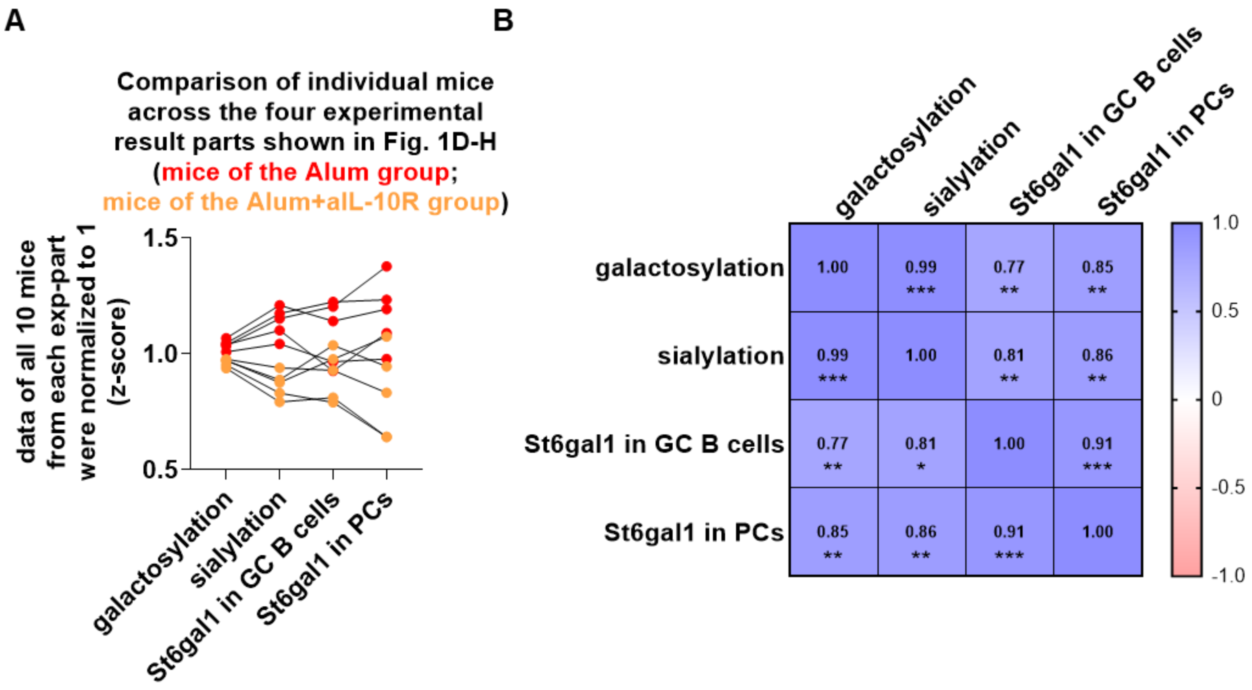

Supplementary Fig. S1: Comparison of individual mice across the four experimental readouts shown in Fig. 1D-H.

(A) For visualization, data from each experimental readout in **Fig. 1D-H** were normalized across all 10 mice using Z-score transformation (mean = 1). For **Fig. 1E**, agalactosylation (G0) values were converted to galactosylation values (100% − G0%) prior to normalization. Data points from individual mice are connected by lines.

(B) Pearson correlation analysis of the normalized data shown in (A). Pearson correlation coefficients (r) values and corresponding p-values are indicated. *$p < 0.05$, **$p < 0.01$, ***$p < 0.001$.

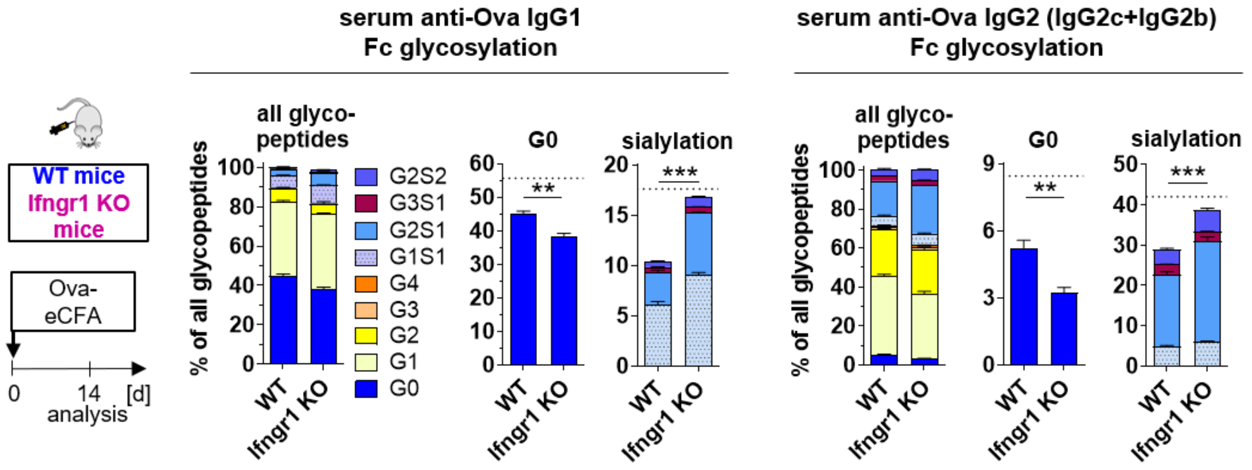

Supplementary Fig. S2: IFNγ downregulates IgG galactosylation and sialylation.

A presentation of previous published data (E12 (3)). C57BL/6 WT (n=10) and *Ifngr1*-KO (n=5) mice were immunized i.p. with Ova together with eCFA and analyzed on day 14 for serum anti-Ova IgG1 and IgG2 (IgG2c + IgG2b) Fc glycosylation, including agalactosylation (G0), and accumulated sialylation (G1S1F + G2S1F + G3S1F + G2S2F), as determined by nLC-MS (percentages, mean ± SEM). Significances are based on two-tailed t tests (*p < 0.05, **p < 0.01, ***p < 0.001). The Shapiro–Wilk test revealed no significant deviation from normal distribution in any of the datasets. One of two independent experiments is shown.

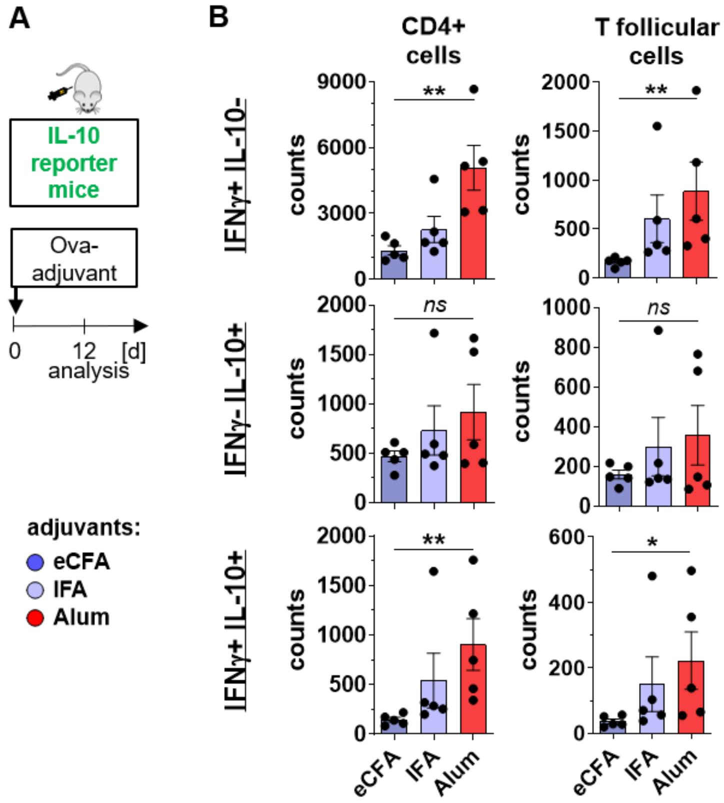

Supplementary Fig. S3: Counts of IFNγ^{+} IL-10^{+} T follicular cells upon immunization with different adjuvants.

Data from the same experiment as shown in **Fig. 2C-E**.

(A) Experimental design: IL-10 reporter (GFP, green fluorescent protein) mice were immunized i.p. with Ova together with the indicated adjuvants, and analyzed on day 12 using flow cytometry (~1.4 million splenocytes per sample; n = 5 per group).

(B) Total counts of (i) IFNγ^{+} IL-10^{-}, (ii) IFNγ^{-} IL-10^{+}, and (iii) IFNγ^{+} IL-10^{+} within CD4^{+} cells and CD4^{+} ICOS^{+} CXCR5^{+} T follicular cells are shown for all mice in bar graphs (mean ± SEM). Significances are based on non-parametric Kruskal–Wallis tests with Dunn's post hoc multiple comparisons tests. *$p < 0.05$, **$p < 0.01$, ***$p < 0.001$. One of two independent experiments is shown.

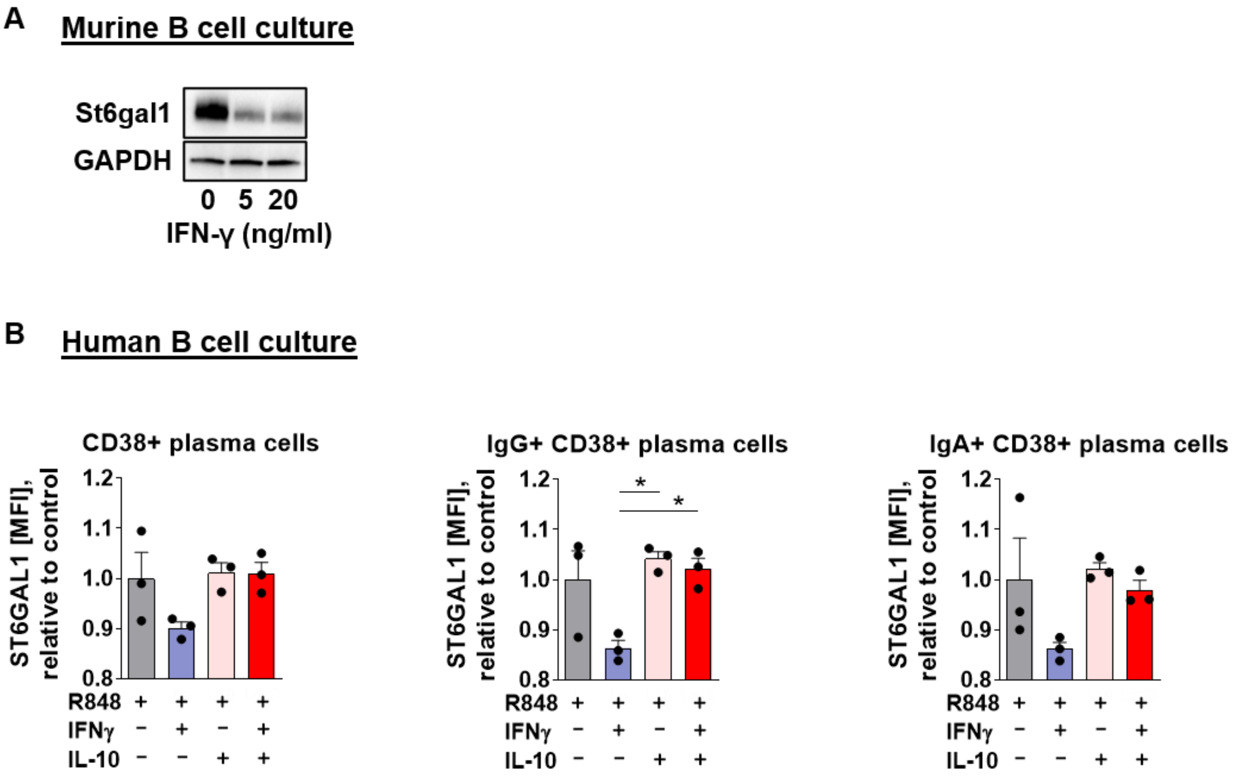

Supplementary Fig. S4: Murine and human B cell cultures.

(A) Repeat of the murine B cell culture with different concentrations of IFNγ, as shown in Fig. 2G. Experimental design: enriched splenic murine B cells from untreated mice were stimulated with LPS and the indicated concentrations of IFNγ and analyzed on day 4. St6gal1 and GAPDH protein expression in cultured cells determined by Western blotting; pooled samples from each group were loaded.

(B) Human B cell culture. Experimental design: Human purified PBMCs were stimulated with R848 and 10 ng/mL IFNγ and/or 100 ng/mL IL-10 and analyzed on day 5. Median fluorescence intensity (MFI) of ST6GAL1 protein expression in $CD38^+$, IgG^+ $CD38^+$ and IgA^+ $CD38^+$ PCs measured by flow cytometry (n = 3 per group). Data are shown as mean ± SEM and were normalized to the R848-only group. Two-way ANOVAs with Tukey's post hoc multiple comparisons tests were used for statistical analysis because of two (cytokine) factors. $*p < 0.05$, $**p < 0.01$, $***p < 0.001$.
